# Epithelioid sarcoma and its outcome: a retrospective analysis from a tertiary care center in North India

**DOI:** 10.2144/fsoa-2021-0138

**Published:** 2023-01-10

**Authors:** Divya Kashyap, Sameer Rastogi, Vikas Garg, Shakti Shrivastava, Adarsh Barwad, Shamim A Shamim, Angel Hemrom, Ekta Dhamija, Sandeep Bhoriwal, Rakesh Garg

**Affiliations:** 1 Department of Medical Oncology, AIIMS, New Delhi, 110029, India; 2 Department of Pathology, AIIMS, New Delhi, 110029, India; 3 Department of Nuclear medicine, AIIMS, New Delhi, 110029, India; 4 Department of Radiology, AIIMS, New Delhi, 110029, India; 5 Department of Surgical oncology, AIIMS, New Delhi, 110029, India; 6 Department of Onco-Anesthesia & Palliative Medicine, AIIMS, New Delhi, 110029 India

**Keywords:** chemotherapy, doxorubicin, epithelioid sarcoma, immunotherapy, INI1, overall survival, partial response, pazopanib, soft tissue sarcoma, tazemetostat

## Abstract

**Aim:**

Clinicopatholgical findings and outcomes in epithelioid sarcoma (ES) patients.

**Materials & methods:**

ES patients registered in sarcoma clinic from 2015 to 2021.

**Results:**

There were 20 patients with median age of 26 years. Majority had distal ES (70%) and advanced disease (85%). In patients with advanced disease lymph nodes were involved in 65%, lungs in 58% and others in 35%. Among 14 patients who underwent biopsy outside our institute, nine (64.2 %) had been initially misdiagnosed. Response rates to doxorubicin (n = 12), pazopanib (n = 6), gemcitabine/docetaxel (n = 5), tazemetostat (n = 3) and immunotherapy (n = 2) used in various lines were 16, 16, 20, 33 and 0%, respectively.

**Conclusion:**

Our patients had an advanced-stage and distal ES, with a modest response to chemotherapy.

## Plain language summary

Epithelioid sarcoma is an uncommon subtype of soft tissue sarcoma with a variable clinical course. We analyzed the outcomes of 20 patients registered under our Sarcoma Medical Oncology clinic from 2015 to 2021. The majority of our patients had an advanced stage at presentation with lymph nodal and lung metastasis. Due to rarity and overlapping histological findings many patients may be initially misdiagnosed. Patients with advanced stages are treated with various chemotherapeutic agents, which have very low response rates. Tazemetostat has shown some promise with responses in up to a third of patients.

Epithelioid sarcoma (ES) is an exceedingly rare, slow-growing neoplasm that was first described by FM Enzinger in 1970 [1]. It constitutes only 1% of all soft tissue sarcomas. There are two distinct subtypes of ES, namely, classic ES, which typically presents as a subcutaneous or deep dermal mass in the distal extremities of the young and the proximal variant, which has a propensity for proximal part of limbs, limb girdles and the midline of the trunk [2].

Histopathologically, these two subtypes exhibit distinct morphology. The classic ‘distal’ form of ES shows a characteristic nodular growth pattern. The tumor cells are a mixed population of eosinophilic epithelioid and spindle cells exhibiting mild nuclear atypia. The center of the tumor nodules undergoes necrosis with peripheral preserved tumor cells resulting in a pseudo granulomatous appearance on low power magnification. But there is mitotic activity with presence of few atypical mitotic figures [3]. Proximal-type ES is found mainly in the pelvic, perineal regions and genital tracts. Microscopically, it has a multinodular pattern of growth, consists of large epithelioid carcinoma-like cells with marked cytological atypia, vesicular nuclei and prominent nucleoli. There is the frequent occurrence of rhabdoid features in the tumor cells and the absence of a granuloma-like pattern [4,5]. Additionally, these cells express cytokeratin, epithelial membrane antigen and endothelial markers like CD34 and ERG in around 50–60% of cases [6,7]. Both classic and proximal type of ES shows consistent loss of expression of INI1 (HSNF5/SMARCB1) by immunohistochemistry [8]. This can also be observed in malignant renal and extra-renal rhabdoid tumors, epithelioid malignant peripheral nerve sheath tumors, poorly differentiated chordoma, extra skeletal myxoid chondrosarcoma and myoepithelial carcinoma [9].

The clinical behavior of ES is quite unpredictable. Some patients have no recurrence of their disease, whereas others have a slow, relentless progression with multiple local recurrences and distant metastatic spread. The most important prognostic factors in epithelioid sarcoma are age, anatomical site, grade, tumor, nodes, and metastases (TNM) staging and treatment modality. Younger patients and patients with distal locations have better outcomes while proximal type epithelioid sarcoma had a poorer prognosis [10,11].

The outcome of metastatic ES is dismal due to its chemo insensitive nature. Response rates to gemcitabine-based regimen and doxorubicin-based regimens are 27 and 22%, respectively, while responses are rarely observed with pazopanib group. The median progression-free survival (PFS) in gemcitabine-based chemotherapy, doxorubicin-based chemotherapy and pazopanib was 6, 4 and 3 months, respectively [11]. Recently, tazemetostat has been approved by US FDA for treatment of advanced ES. In a phase II basket study the response rate of tazemetostat was 15% while PFS was 5.5 months (95% CI: 3.4–5.9) [12].

So far, the data about ES and its outcome is sparse in India. This report aims to describe the clinical pathological profile and outcomes of our patients on various regimens including tazemetostat.

## Materials & methods

It is a retrospective study evaluating patients with ES registered in a sarcoma medical oncology clinic from January 2015 to June 2021. Prior to the start of the study, approval was obtained from the ethics review board of our institute, All India Institute of Medical Science (New Delhi, India) and the assigned ethical review no. is IEC-565/16 August 21. The pathology of all the cases was reviewed by a sarcoma pathologist, and all cases were discussed in a multidisciplinary clinic. The various chemotherapy regimens used in the clinic depended on the physician’s discretion and tolerance of the patient. Data were studied through hospital records, including the age, sex, site of disease, stage, histopathology, treatment details, response rates and outcomes.

SPSS version 24 was used for statistical analysis. Nominal data are provided as numbers (%), continuous data as median (range) and through charts. PFS was calculated from the date of diagnosis or subsequent change of therapy to the first date of documented progressive disease or the date of death from any cause. Overall survival (OS) was calculated from the date of diagnosis to the date of death or the date of the last follow-up.

## Result

**Figure 1.**
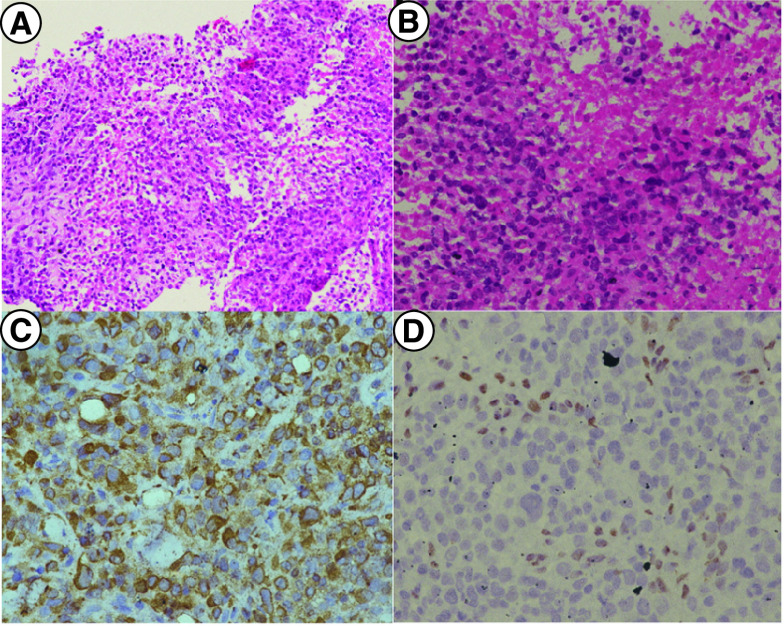
Histopathology and immunohistochemistry of one of our patients with epithelioid sarcoma. **(A)** Low power histomicrophotograph of the tumor showing tumor cells in syncytium and nests with central necrosis. **(B)** H&E 100×High power showing tumor cells morphology which is epithelioid shaped with vesicular chromatin and conspicuous nucleoli. The cytoplasm is abundant and eosinophilic. The central portion show coagulative necrosis of the tumor cells. **(C)** H&E 200× Immunostain for cytokeratin showing diffuse cytoplasmic immunoreactivity, **(D)** with diffuse nuclear loss of INI1 expression.

There were a total of 20 patients with ES registered in our clinic from January 2015 to June 2021. Among these patients, 14 (70%) presented with distal-type ES and 06 (30%) had proximal-type ES. The median age was 26 years (range: 9–60 years), including three pediatric patients. There were 11 (55%) males and 9 (45%) females. Among females, two were pregnant at the time of diagnosis and one patient was diagnosed 2 months post pregnancy. The baseline characteristics of patients are depicted in Table 1. Of 14 patients who had been biopsied outside (n = 14), nine patients had different diagnoses from the outside center. After review by an expert sarcoma pathologist, these were diagnosed as ES. The discrepancy in the pathological findings is summarized in Table 2. Figure 1 shows the histopathology details of a patient who was initially misdiagnosed with epithelioid leiomyosarcoma. On review of pathology and immunohistochemistry, the diagnosis of ES was confirmed by a sarcoma pathologist.

**Table 1.** Baseline characteristics of patients with epithelioid sarcoma.

Patient characteristics	n = 20 (%)
Median age (range)	26 (9–60 years)
Performance status • ≤2 • >2	17 (85%) 3 (15%)
Sex • Male • Female	11 (55%) 9 (45%)
Pediatric patients	3 (15%)
During pregnancy and post partum	3 (15%)
Type of tumor • Proximal • Distal type	6 (30%) 14 (70%)
Disease status at presentation • Metastatic • Non metastatic	17 (85%) 3 (15%)
Site of metastasis (n = 17) • Lymph node • Lung • Lymph node and lung • Others	11 (65%) 10 (58%) 5 (29%) 6 (35%)
Total number of surgeries performed (n = 34) • WLE • Amputation • Excisional biopsy	26 (76%) 6 (18%) 2 (6%)

WLE: Wide local excision.

**Table 2.** Discrepancy in the histopathology reports of patients.

Outside biopsy	Expert histopathology
Synovial sarcoma(n = 2)	Epithelioid sarcoma
Epithelioid leiomyosarcoma (n = 1)	Epithelioid sarcoma
Alveolar soft part sarcoma/ melanoma (n = 1)	Epithelioid sarcoma
Squamous cell carcinoma (n = 1)	Epithelioid sarcoma
Myoepithelioma (n = 1)	Epithelioid sarcoma
Soft tissue sarcoma NOS (n = 2)	Epithelioid sarcoma
Epithelioid angiosarcoma (n = 1)	Epithelioid sarcoma

A total of 34 surgeries (median surgeries [n]: 2 [range: 0–4]) were performed in the study populations with the majority (95%, n = 19) of patients undergoing at least one surgical intervention. Among these wide local excisions, amputation and excisional biopsy was 26 (76%), 06 (18%) and 02 (6%) respectively. Data regarding surgical margin was available for only 12 patients as most patients underwent surgeries at other centers. Margins were free from a tumor in 11 (91.6%) and positive in 1 (8.3%) patient.

**Figure 2.**
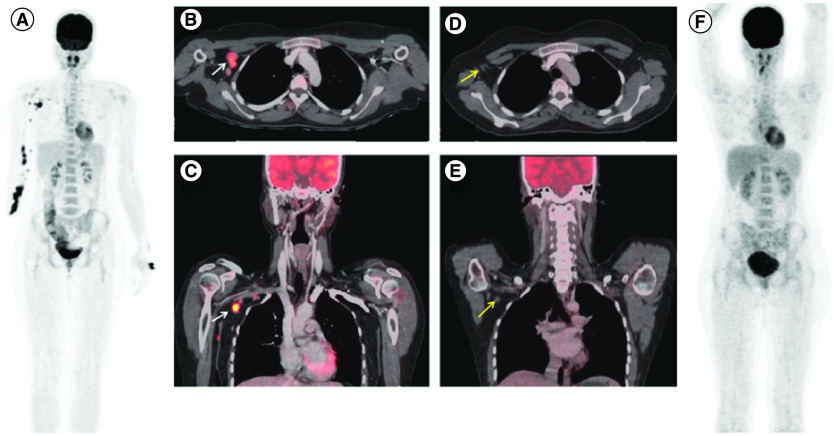
A 29-year-old female presented with history of recurrent soft tissue mass in right hand and had multiple surgeries. ^18^F-FDG positron emission tomography/computed tomography (PET/CT) scan was performed in August 2020. **(A)** MIP image showed multiple FDG avid soft tissue density deposits in subcutaneous and muscular planes of right upper limb along with right axillary lymph nodes. **(B)** Axial and **(C)** coronal cross section images showed enlarged FDG avid right axillary lymph nodes (white arrows). After chemotherapy, 18F-FDG PET/CT was done to assess response in May 2021. **(D)** Axial and **(E)** coronal images shows reduction in size of axillary lymph nodes (yellow arrow) with no significant FDG uptake. **(F)** Maximum intensity projection image showed metabolic resolution of lesions previously observed, suggesting complete metabolic response.

All patients with advanced ES were started on systemic therapy. Doxorubicin, pazopanib, gemcitabine with docetaxel, tazemetostat and immunotherapy-based regimen was administered to 12 (63%), 6 (32%), 5 (26%), 3 (16%) and 2 (11%) patients, respectively, as shown in Table 3. There were no grade 3 or 4 toxicity found in people receiving gemcitabine with docetaxel, tazemetostat and immunotherapy. However, doxorubicin-based regimen had grade 3/4 neutropenia in 2/12 (17%), anemia in 2/12 (17%) and vomiting 1/12 (8%). Pazopanib grade 3 diarrheas were present in 2/6 patients (33%).The best response to the doxorubicin-based regimen was partial response in 02 (16%) patients, gemcitabine with docetaxel had partial response in 01 (20%) patient, pazopanib had partial response in 01 (16%) and tazemetostat had partial response in 01 (33%). No objective response was observed in patients who received immunotherapy. Figure 2 depicts 18F-FDG PET/CT images of a patient before and after chemotherapy.

**Table 3.** Details of systemic therapy administered in epithelioid sarcoma patients.

Type of chemotherapy used in different lines	Frequency n (%)	Response rate n (%)
Doxorubicin based Gemcitabine with docetaxel Pazopanib Tazemetostat Immunotherapy	12 (63%) 05(26%) 06(32%) 03(16%) 02(11%)	2 (16%) 01 (20%) 01 (16%) 1 (33%) 0

**Figure 3.**
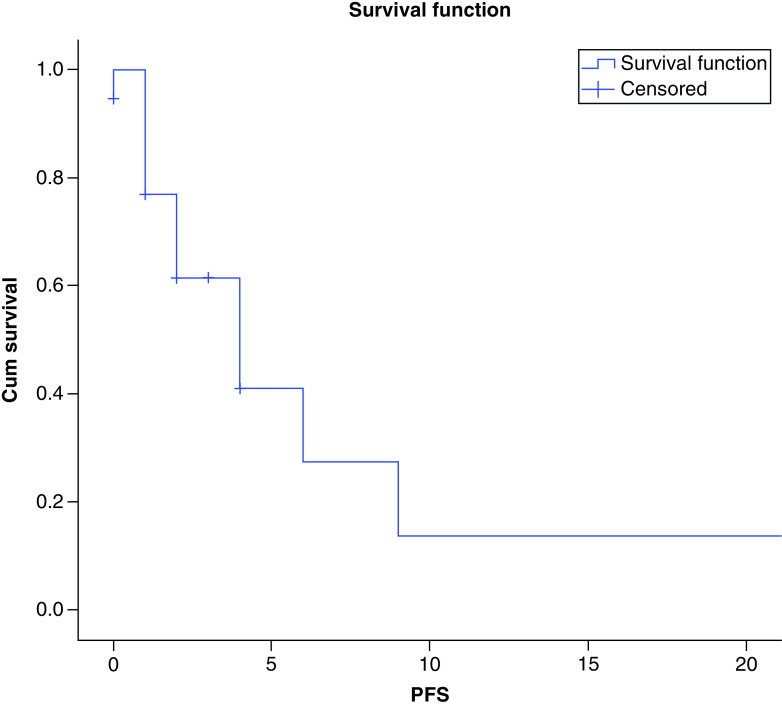
Progression free survival in patients with epithelioid sarcoma.

**Figure 4.**
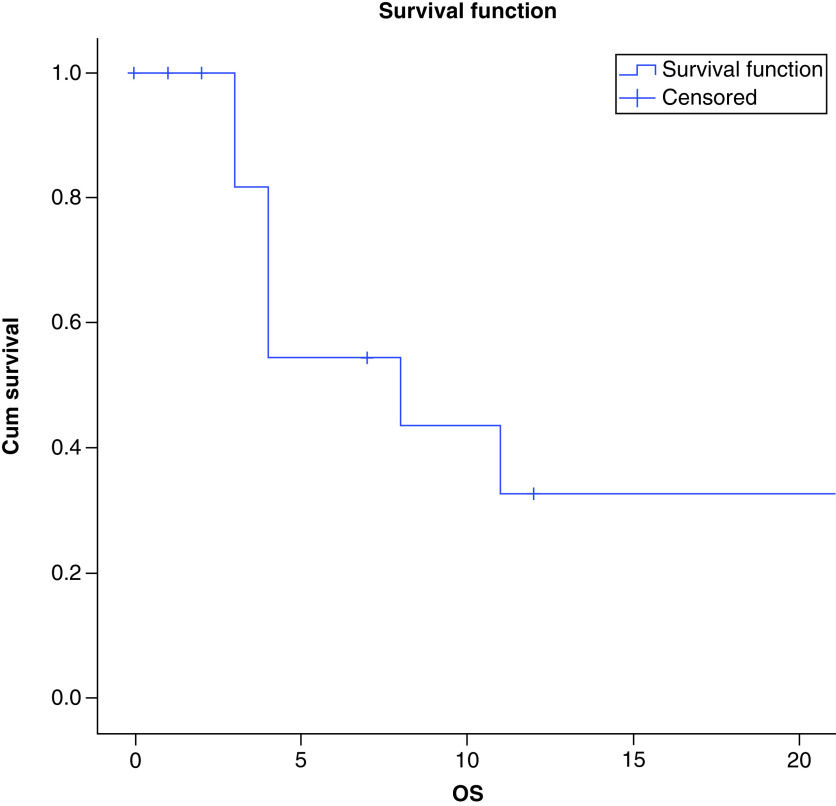
Overall survival in patients with epithelioid sarcoma.

There were a total of seven deaths recorded in our series. At a median follow-up of 12 months, the median PFS of the patients in our study was 4 months and the median OS was 8 months as shown in Figures 3 & 4, respectively. There was no difference between the survival of metastatic and non metastatic patients (p = 0.3), and could be attributed to low numbers of non-metastatic patients.

## Discussion

Epithelioid sarcoma outcomes have been rarely reported from developing countries [13]. The median age in our study was 26 years reflecting a distinctly younger population as compared with western studies [14]. This could be partly due to the inclusion of pediatric patients as well. Male predominance is similar to that described in the literature [15].

There are few cases reported during pregnancy and post partum vulvar epithelioid sarcoma; however, in our series all three patients during pregnancy and post partum epithelioid sarcoma had non-vulvar primary site. Among eight females in our study, two were pregnant at the time of diagnosis and one patient presented 2 months after delivery. Previous reports do not indicate any relation between pregnancy and tumor growth [13,14]. Our study included about 15% pediatric patients, which has been rarely reported in the literature [15,16].

The pathological discrepancy after review by an expert sarcoma pathologist is observed in upto two third of cases as seen in this study. There is plenty of literature to show that difficulty in diagnosing this tumor stems from both rarity and resemblance to other sarcomas [17–20].

Majority of patients had metastatic disease at presentation, and only three patients had no metastatic disease sites. This could be due to a selective referral pattern in sarcoma medical oncology clinics. In our cohort, proximal epithelioid sarcoma was less common and constituted less than a third of cases. The ratio of proximal to distal varies in the literature. In a series from India, the proximal type was almost twice as prevalent as the distal type [2]. On the other hand, the proximal subtype is almost equal to distal subtype in western population [12]. This discrepancy could be attributed to either more prevalence of distal subtype in India or to referral bias.

Lymph node metastasis is the most common site of metastasis with two third patients having lymph node involvement in our series. In literature, lung is the most common site of metastasis and lymph node involvement is observed in around 30–45% cases [11]. However, in our series lymph node involvement is slightly more common than lung metastasis.

 Chemotherapy outcomes in epithelioid sarcoma have been mainly retrospective due to conspicuous rarity and lack of standard treatment. In a retrospective analysis by Jones *et al.*, 21 patients with ES who received chemotherapy between 1990 and 2009, the response rate to palliative chemotherapy was 15% and median PFS was 29 weeks [21]. Furthermore, only seven and three patients could receive second- and third-line chemotherapy, respectively. The most common chemotherapy used in these reports was single-agent doxorubicin followed by combination therapy. Similarly, in the report by Frezza *et al.*, the chemotherapy response rates with gemcitabine-based regimen and doxorubicin-based regimens were 27 and 22%, respectively. The median (PFS) in gemcitabine-based chemotherapy and doxorubicin-based chemotherapy was 6 and 4 months, respectively [11]. No partial remission was seen with pazopanib [11]. In the study by Gounder *et al.*, of the 62 patients receiving tazemetostat for advanced epithelioid sarcoma, response was observed in nine (15%) patients, and it was well tolerated [12]. Similar responses were observed in our study. Responses were observed in one-fourth of patients receiving palliative systemic therapy with highest response rate to tazemetostat and lowest to immunotherapy. Most patients were started on single agent doxorubicin and responses were seen in one sixth of patients. OS and PFS were 8 and 4 months with a median follow-up of 12 months.

In our cohort two (11%) patients received immunotherapy; however, none of them responded. Literature is sparse about the role of immunotherapy in epithelioid sarcoma as compared with another sarcoma like undifferentiated pleomorphic sarcoma (UPS) and alveolar soft part sarcoma (ASPS). In a report by Pecora *et al.*, a 19-year-old male who had metastatic epithelioid sarcoma refractory to multiple therapies including tazemetostat, had a complete response on nivolumab and ipilimumab therapy [22].

## Conclusion

Epithelioid sarcoma is a rare entity. High rate of pathologic discordance in diagnosis is seen when reported by unexperienced pathologists. Majority patients had advanced disease and the most common site of metastasis was lymph nodes and lung. It is a disease of young people, and the distal type of epithelioid sarcoma is most common subtype. Chemotherapy and immunotherapy are not very effective and have poor outcomes.

Epithelioid sarcoma is a relatively chemo-refractory disease and available agents have very modest efficacy. Further efforts should be made to explore the biological pathways of carcinogenesis in this entity, so that targeted therapies may be introduced. Multinational collaborative projects may pave way for better understanding and outcomes in this disease.

**Summary points**

- Epithelioid sarcoma (ES) is an uncommon slow growing neoplasm with unpredictable course.
- Due to rarity and overlapping histological features with other malignancies, diagnosis may be missed by unexperienced pathologists.
- It is commonly diagnosed in young people (median age 26 years) but may also be seen in children (15% in this series).
- Most patients (two-thirds) have advanced disease at presentation with lung and lymph node involvement.
- Two histological subtypes, classic or distal and proximal have been named on basis of location of the tumor. On immunohistochemistry, ES shows loss of expression of INI1 (*hSNF5*/*SMARCB1*).
- Multiple chemotherapeutic and targeted agents have been tried in the past including doxorubicin, gemcitabine and docetaxel and pazopanib. However, responses are seen in not more than one-fifth of patients.
- Recently tazemetostat and immunotherapy have also been brought into armamentarium against ES. Tazemetostat has shown good promise with responses in up to one third of patients and has good tolerability. However, there are only case reports of patients responding to immunotherapy. In our study no response was seen in two patients receiving immunotherapy.
- Further collaborative research is warranted to improve the outcomes in ES.
